# Furuncular Myiasis of the Scalp

**Published:** 2016-05-25

**Authors:** Saptarshi Biswas, Patrick McNerney

**Affiliations:** Department of Trauma and Acute Care Surgery, Forbes Regional Hospital, Allegheny Health Network, Pittsburgh, PA

**Keywords:** furuncular myiasis, maggots, Diptera flies, Rodent botfly, larvae

**Figure:**
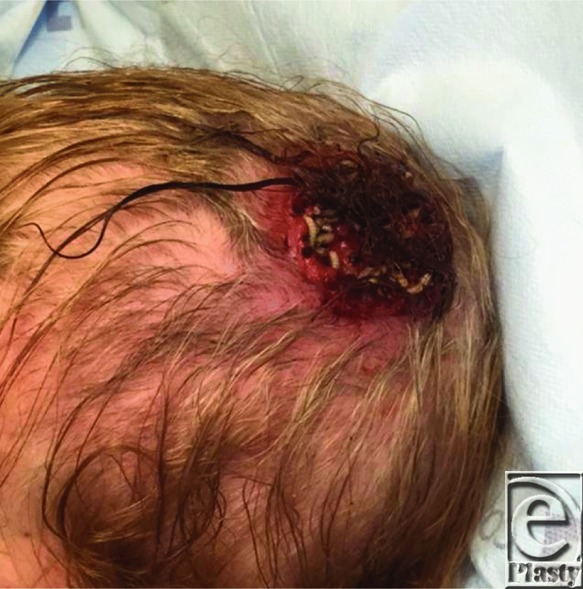


**Figure:**
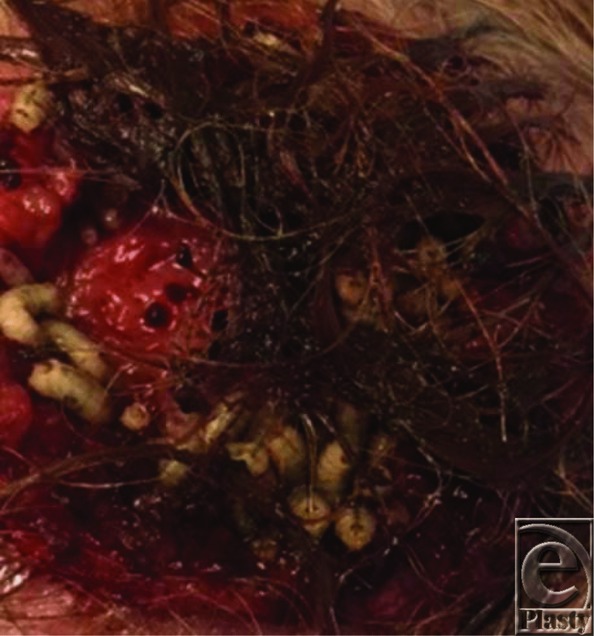


## DESCRIPTION

A 90 year old woman presented to the ED after a fall and bleeding into a scalp wound mimicking pyoderma. She also noticed crawling larvae in the wound.

## QUESTIONS

1. **What was the patient's diagnosis?**
2. **What is myiasis?**
3. **What species are involved in myiasis?**
4. **What treatments exist for this condition?**

## DISCUSSION

This is a case of furuncular myiasis, a human infestation of maggots of the Diptera flies. It is a type of cutaneous myiasis.

The term “myiasis” defines a series of diseases caused by infestation via larvae of the order Diptera. The forms of myiasis are generally differentiated on the basis of clinical and entomological presentation.^1^ Classification of myiasis on entomological grounds typically results in 3 groups: facultative, obligatory, and accidental/pseudomyiasis.^1^ Classification on clinical grounds is based on anatomical location of the infestation.^1^ The locations determining clinical classification are cutaneous (furuncular and wound), sanguinivorous (bloodsucking), migratory, and cavitary myiasis.^1^ Maggot therapy (also called maggot debridement therapy, larvae therapy, biodebridement, or biosurgery) is a type of biotherapy that involves the introduction of live, disinfected maggots (fly larvae) into the nonhealing wounds for cleaning out the necrotic tissue and disinfection. The Food and Drug Administration in 2004 cleared maggots for use as a medical device in the United States for the purpose of treatment of nonhealing necrotic skin and soft-tissue wounds, nonhealing traumatic or postsurgical wounds, pressure ulcers, neuropathic foot ulcers, and venous stasis ulcers.

Although a number of larvae species are found to be associated with myiasis, *Cuterebra* species, the rodent botfly, has a well-known association with furuncular myiasis.^1^ *Dermatobia hominis*, human botfly, however, is the most common cause of furuncular myiasis in the continents of North America and South America.^1^ The presence of malignancy suggests infestation with larvae from *Calliphoridae* and *Sarcophagidae*, as they are almost exclusively associated with infestation in cases of malignancy.^1^ *Lucilia sericata*, also known as the green bottle fly, is the most common species identified in malignant wound maggot infestations.^1^ *Lucilia sericata* has a common distribution in temperate and tropical areas throughout the world, particularly the southern hemisphere.^2^ Females will lay eggs in necrotic tissue, usually in batches of 150 to 200.^3^ The eggs will hatch in around 9 hours, and the larvae released will feast on necrotic tissue for 3 to 10 days before transforming into pupae and falling off their host.^3^ The pupae will then develop in the soil for another 6 to 14 days until they develop into full-fledged adults, going on to reproduce and perpetuating the aforementioned life cycle.^3^ No taxonomic studies were conducted on the larvae.

Treatment of myiasis involving an open wound requires the physical removal of all visible larvae.^4^ Treatment can be further complicated if larvae begin to tunnel away from the wound; for this reason, other topical remedies are often used. In some cases, 15% chloroform in olive oil has been used to prevent tunneling, as it is postulated to paralyze larvae.^1^ Other topical therapies include a solution of 1% ivermectin in propylene glycol.^5^ Use of per os medications for myiasis (eg, ivermectin) is not preferred.^1^ If this lesion had not been open, the furuncular nature would likely suggest an initially more delicate approach involving topical ointments, such as polymyxin B and ivermectin solution; also occluding substances, such as adhesive tapes and petrolatum, are used to create localized hypoxia.^1^ These measures will kill the larvae. Before death, in an attempt to escape the toxic/hypoxic conditions present in the area, the larvae will often emerge from the wound, allowing for a less complicated removal.^6^ Surgical removal is occasionally required if the previously mentioned methods are unsuccessful.^7^ Removal of larvae must be done with utmost care, as leaving fragments of larvae within the wound are associated with postprocedural/operational infections.^7^ Treatment of the remaining wound post-larvae removal is surgical debridement of the necrotic tissue and, in this case, potentially malignant tissue.^4^ Debridement is generally followed by pressure irrigation using normal saline.^1^ Further complex wound closures may be warranted on the basis of the particular shape and size of the wound, including skin grafting.
